# Editorial: Novel emulsion systems: Solutions to enhance nutrient bioavailability and healthy diets

**DOI:** 10.3389/fnut.2022.1074076

**Published:** 2022-12-07

**Authors:** Aimin Shi, Hongzhi Liu, Jin-Rong Zhou, Benu Adhikari, Dominic Agyei

**Affiliations:** ^1^Institute of Food Science and Technology, Chinese Academy of Agricultural Sciences, Beijing, China; ^2^Key Laboratory of Agricultural Product Processing and Quality Control, Ministry of Agricultural and Rural Affairs, Beijing, China; ^3^Beth Israel Deaconess Medical Center, Harvard Medical School, Boston, MA, United States; ^4^School of Science, RMIT University, Melbourne, VIC, Australia; ^5^Department of Food Science, University of Otago, Dunedin, New Zealand

**Keywords:** emulsion systems, nutrient bioavailability, delivery system, future food, precise nutrition

This research theme aims to highlight latest research findings on the development and design of new emulsion systems and provide greater insight on the mechanism involved in their development and function. It also aims to highlight their application in food processing and ways to improve bioavailability of functional components and making these new emulsion systems as part of healthy diet.

Pickering emulsions, which are stabilized by particles, have gained considerable attention recently because of their high degree of stability and functionality. Food-grade Pickering particles are preferred by food or pharmaceutical industries due to their additional benefits (renewable resource, ease of preparation, biocompatibility, and unique interfacial properties). In the review titled “Recent Advances on Pickering Emulsions Stabilized by Diverse Edible Particles: Stability Mechanism and Applications” systematically summarizes mechanism involved in stability of Pickering emulsion, especially the nature of capillary pressure between particles, the interfacial rheological response, and the formation of particles' network in the space between droplets (Li et al.). The effects of particle shape and concentration, pH, concentration, ionic strength, temperature, and oil phase on the stability of Pickering emulsion are systematically analyzed. Pickering emulsions are now widely used in cosmetic, pharmaceutical, tissue engineering, and food fields due to their many desirable properties.

When the oil phase is >74%, the emulsion stabilized by solid Pickering particles is also called “high internal phase Pickering emulsion” (HIPPE). There are many advantages of HIPPE: less dosage of stabilizer, high stability of anti-coalescence, good storage stability, and small environmental pollution. Protein-based particles are promising as HIPPE stabilizers because they do not require additional surface modification and are easy to process. This paper “Characterization of a Novel High Internal Phase Pickering Emulsions Stabilized by Soy Protein Self-Assembled Gel Particles” reports a novel HIPPE prepared from acid-induced self-assembled SPI gel (A/S-SPIG; Bi et al.). The potential of A/S-SPIG particle as an efficient HIPPE stabilizer is studied from the perspectives of rheology, thermodynamics, and microstructure. The result show that gradually increasing the concentration of A/S-SPIG particles in the emulsion system strengthens the elasticity and viscosity properties of HIPPE. The increase of A/S-SPIG particle concentration has shown to effectively improve the emulsification effect of HIPPE, as indicated by the gradual decrease of mean particle size of emulsified droplets. The proposed optimal concentration of A/S-SPIG particles is shown to improve the freeze-thaw stability of HIPPE.

It has been shown that protein-protein complexes can be formed by electrostatic attraction, which can improve the stability of emulsions and also the stability of oils against oxidation. The stability of soy protein isolate (SPI)/bamboo shoot protein concentrate (BPC) complex on oil-in-water (O/W) camellia oil emulsion was studied in “Improved Oxidation Stability of Camellia Oil-in-Water Emulsions Stabilized by the Mixed Monolayer of Soy Protein Isolate/Bamboo Shoot Protein Complexes” (Xi et al.). The hydrogen bonding and electrostatic are shown to dominate the binding of BPC to SPI, which improved the surface hydrophobicity and induced a network layer structure of the SPI/BPC complex. With the increase of the proportion of BPC in the complex, the resulting complex formed a compact network layer structure due to the rearrangement of proteins, and the gelatinous structure of the emulsion was gradually enhanced, showing better emulsification activity and stability. Moreover, in paper “Water-Dispersible Phytosterol Nanoparticles: Preparation, Characterization, and *in vitro* Digestion,” phytosterols (PS) nanoparticles with soy protein isolate (SPI) and soybean lecithin (SL) were successfully fabricated by emulsification-evaporation combined high-pressure homogenization method (Li et al.). The results suggest that this is an effective approach for creating food-grade PNPs with good water dispersibility. Compared with the raw PS, the incorporation of SL with PS in PNPs showed remarkable improvement of bio-accessibility.

The fat substitutes made from different proteins or polysaccharides are added to various meat products, including sausages and patties. However, few studies have involved 3D structural optimization of the simulated pork fat products. The paper titled “Create Fat Substitute from Soybean Protein Isolate/Konjac Glucomannan: The Impact of the Protein and Polysaccharide Concentrations Formulations” reports that soybean protein isolate (SPI) and coconut oil were used as emulsifiers, and konjac powder was added to prepare protein/polysaccharide composite emulsion gel (Huang et al.). SPI/polysaccharide composite as a fat substitute was obtained by a simple vacuuming. This novel composite is shown to better mimic the appearance and taste of natural pork fat without affecting mechanical, rheological, and thermal properties. Solid fat substitutes prepared with SPI and konjac powder were similar in appearance to pork fat. These fat replacers show ideal functional properties in terms of mechanical properties and oral tribology.

In summary, this research theme focuses on *Novel Emulsion Systems: Solutions to Enhance nutriment Bioavailability and Healthy Diets*, includes one review and four research articles. These articles demonstrate the design and synthesis of novel emulsion systems possessing improved bioavailability of functional components and their application in healthy diets.

## Author contributions

AS, HL, J-RZ, BA, and DA are responsible for the management of the whole issue. All authors contributed to the article and approved the submitted version.

